# CHAC1 Is Differentially Expressed in Normal and Cystic Fibrosis Bronchial Epithelial Cells and Regulates the Inflammatory Response Induced by *Pseudomonas aeruginosa*

**DOI:** 10.3389/fimmu.2018.02823

**Published:** 2018-11-29

**Authors:** Léa Perra, Viviane Balloy, Tobias Foussignière, Didier Moissenet, Hortense Petat, Imran N. Mungrue, Lhousseine Touqui, Harriet Corvol, Michel Chignard, Loic Guillot

**Affiliations:** ^1^Sorbonne Université, Inserm, Centre de recherche Saint-Antoine (CRSA), Paris, France; ^2^Department of Bacteriology, APHP, Hôpital St-Antoine, Paris, France; ^3^Department of Pharmacology and Experimental Therapeutics, Louisiana State University Health Sciences Center, New Orleans, LA, United States; ^4^Equipe mixte Institut Pasteur/Paris V “Mucoviscidose et Bronchopathies Chroniques” Institut Pasteur, Paris, France; ^5^Pneumologie Pédiatrique, APHP, Hôpital Trousseau, Paris, France

**Keywords:** cystic fibrosis, bronchial epithelium, *Pseudomonas aeruginosa*, inflammation, ChaC glutathione-specific γ-glutamylcyclotransferase 1, ER stress

## Abstract

In cystic fibrosis (CF), *Pseudomonas aeruginosa* (*Pa*) colonizes the lungs, leading to chronic inflammation of the bronchial epithelium. ChaC glutathione-specific γ-glutamylcyclotransferase 1 (*CHAC1*) mRNA is differentially expressed in primary human airway epithelial cells from bronchi (hAECBs) from patients with CF and healthy patients at baseline and upon infection with *Pa*. CHAC1 degrades glutathione and is associated with ER stress and apoptosis pathways. In this study, we examined the roles of CHAC1 in the inflammatory response and apoptosis in lung epithelial cells. First, we confirmed by reverse transcription quantitative polymerase chain reaction that *CHAC1* mRNA was overexpressed in hAECBs from patients without CF compared with the expression in hAECBs from patients with CF upon *Pa* (PAK strain) infection. Moreover, the *Pa* virulence factors LPS and flagellin were shown to induce *CHAC1* expression in cells from patients without CF. Using NCI-H292 lung epithelial cells, we found that LPS-induced *CHAC1* mRNA expression was PERK-independent and involved ATF4. Additionally, using CHAC1 small interfering RNA, we showed that reduced *CHAC1* expression in the context of LPS and flagellin stimulation was associated with modulation of inflammatory markers and alteration of NF-κB signaling. Finally, we showed that *Pa* was not able to induce apoptosis in NCI-H292 cells. Our results suggest that CHAC1 is involved in the regulation of inflammation in bronchial cells during *Pa* infection and may explain the excessive inflammation present in the respiratory tracts of patients with CF.

## Introduction

Cystic fibrosis (CF), the most common severe autosomal recessive genetic disease in the Caucasian population, is caused by mutations in the CF transmembrane conductance regulator (*CFTR*) gene (1–3). Lung injury is predominant and is the leading cause of morbidity and mortality in CF patients. In airways, CFTR dysfunction leads to alterations in mucociliary clearance and impairment of innate immune host defenses. This results in chronic bacterial infection and inflammation, leading to degradation of the lung epithelium and progressive lung damage in CF patients (4). *Pseudomonas aeruginosa* (*Pa*) is the leading cause of chronic pulmonary infection in the airways of adults with CF (5). *Pa* colonization induces a strong inflammatory response in CF patients, particularly neutrophil accumulation and hypersecretion of inflammatory cytokines, such as IL-8 (6, 7).

The lung epithelium plays an essential role in the fight against infections by orchestrating innate immunity (8). Lung epithelial cells are sufficient to generate an innate protective immune response in mice infected with *Pa* (9). In CF, establishment of chronic pulmonary infection with *Pa* is linked to alterations in epithelial cell responses, which do not allow establishment of an adequate defense system (4). Previously, we showed that this response was indeed different by comparative analysis of the transcriptomic signatures of primary CF and non-CF bronchial epithelial cells during *Pa* infection (10). We identified several up-and downregulated mRNAs in CF cells compared with those in non-CF cells, suggesting that these genes may be involved in the exacerbated inflammatory response to *Pa* found in CF patients. Among these dysregulated mRNAs, ChaC glutathione-specific γ-glutamylcyclotransferase 1 (*CHAC1*) showed dramatically differential expression between non-CF and CF cells both at baseline and after *Pa* infection.

CHAC1 was identified in mammalian cells for the first time in 2009 as a new component of the unfolded protein response (UPR) pathway (11). This UPR pathway is induced in response to ER stress due to the presence of misfolded proteins in the ER lumen. The UPR consists of three pathways initiated by inositol-requiring enzyme 1α, eukaryotic translation initiation factor 2-α kinase 3 (PERK), and activation transcription factor (ATF) 6 (12). These three pathways alleviate ER stress by decreasing protein synthesis, facilitating protein folding, and increasing protein degradation. CHAC1 is induced by the PERK pathway downstream of ATF4 and the pro-apoptotic factor DNA damage-inducible transcript 3 (DDIT3 also called CHOP) and has been described as necessary and sufficient to induce markers of apoptosis, particularly the cleavage of the apoptotic factor poly(ADP-ribose) polymerase 1 (PARP) (11). Moreover, CHAC1 can degrade glutathione in the cytosol of mammalian cells through its γ-glutamylcyclotransferase activity (13). Various studies have shown a link between ER stress and inflammation (12, 14, 15). In the context of CF, atypical activation of the UPR pathway has been observed; the resulting ER stress can potentiate the inflammatory response (12). Moreover, the UPR is implicated in the regulation of cytokine production for bacterial infections (16). The role of CHAC1 in this interplay between ER stress and inflammation in the context of *Pa* infection has never been investigated.

Accordingly, in this study, we hypothesized that CHAC1 may play a role in regulating the inflammatory process induced by *Pa* infection and apoptosis.

## Materials and methods

### Reagents

*Pa* (serotype 10) LPS (1 μg/ml), MG-132, staurosporine, tobramycine, and DMSO were from Sigma-Aldrich (Saint-Quentin Fallavier, France). Purified *Pa* flagellin (50 ng/ml) was from InvivoGen (San Diego, CA, USA), and Tunicamycine (TM) and GSK2656157/PERK inhibitor were from Calbiochem (Merck Millipore, Molsheim, France).

### Cell culture

NCI-H292 (ATCC-CRL-1848; Rockville, MD, USA), BEAS2-B (ATCC-CRL-9609) and A549-NF-κB luciferase cells (Panomics, Fremont, CA, USA) were grown as previously (17, 18). Primary hAECBs from CF patients or healthy donors (Table 1) were commercially obtained (Epithelix, Plan les Ouates, Switzerland) and were cultured as previously (19). The cells were seeded in different plates (TPP, Techno Plastic Products, Trasadingen, Switzerland) and after reaching confluence and were incubated overnight in DMEM containing 10% FBS and 1% antibiotics before being stimulated in the same medium free of antibiotics.

**Table 1:** Characteristics of patients whose hAECBs were used in this study.

**Patient number**	**Commercial reference**	**Age (years)**	**Sex**	**Smoker**	**Pathology**	***CFTR* mutation**
P1	AB60001	56	F	No	No	No
P2	AB037201	78	M	No	No	No
P3	AB39401	55	M	No	No	No
P4	AB68201	66	F	No	No	No
P7	CF56701	39	F	No	CF	F508del
P8	CF60701	21	F	No	CF	F508del
P9	CF45202	32	M	No	CF	F508del
P10	CF60901	21	F	No	CF	F508del

### Bacterial strains, growth conditions, and infection protocols

The *Pa* WT PAK strain, the mutant ΔFliC with deletion of flagellin and the double mutant ΔpscFΔxcpQ with deletion of type II and III secretion systems were used and grown as previously (20, 21). The OD measured at 600 nm was adjusted to give the desired MOI. MOI were verified by serial 10-fold dilutions of the bacterial suspensions and plating on LB agar (Supplementary Figure 3). Cells seeded in 6-well (hAECBs) or 12-well plates (NCI-H292) were infected with *Pa* in their culture medium without antibiotics. After each stimulation, supernatants were collected and centrifuged at 3,000 × g for 15 min to remove the bacteria. Clinical strains of *Pa* were isolated according to the standards procedures of the microbiological department of St-Antoine Hospital, Paris, France. Sputum from patients was diluted (v/v) with prediluted Digest-Eur (Eurobio, Courtaboeuf, France), vortexed, and incubated for 15 min at room temperature. A 10-μL aliquot was plated on different selective media for 5 days at 37°C under aerobic conditions. *Pa* was isolated from Drigalski's agar plates, and identification was confirmed by mass spectrometry (Maldi-Biotyper, Brucker, France). *Stenotrophomonas maltophilia* was freshly isolated from a CF patient, and its identification was confirmed by mass spectrometry. *S. maltophilia* and *Staphyloccocus aureus* [Newman strain (22)] were cultured using the same protocol for *Pa* as described above.

### CHAC1 and ATF4 inhibition

To induce transient *CHAC1* mRNA inhibition, we performed reverse transfection in NCI-H292 and/or A549 cells with either siRNA for CHAC1 (siRNA CHAC1, s35570) or negative control (siRNA Ctrl) from Ambion (Austin, TX, USA) using HiPerFect transfection reagent (Qiagen, Hilden, Germany). Cells were, respectively, seeded either in 96- (A549) or 12-well (NCI-H292) plates and transfected the same day with 5 nM siRNA. After 48 h of reverse transfection, cells were treated as described in the Figure Legends. To induce transient *ATF4* mRNA inhibition, NCI-H292 cells seeded in 12-well plates were transfected with 5 nM siRNA for ATF4 (s1702, Sigma-Aldrich) or 5 nM negative control siRNA (siRNA Ctrl,) using Lipofectamine 3000 (Invitrogen, Carlsbad, CA, USA) for 48 h.

### RT qPCR

RNA was isolated using a NucleoSpin RNA/Protein kit (Macherey Nagel, Duren, Germany). RT was performed using a high-capacity cDNA kit (Applied Biosystems, Foster City, CA, USA). Real-time qPCR was performed with an ABI StepOnePlus, using TaqMan Fast Universal PCR Master Mix (Applied Biosystems), TaqMan probes for *CHAC1* (Hs_00225520), *IL*-*8* (Hs_174103), *IL*-*6* (Hs_985639), *CCL2* (Hs_00234140), *GAPDH* (Hs_2786624) and cDNA as a template. For relative quantification, the amount of target genes was normalized to the expression of *GAPDH* relative to control cells used as a calibrator and was calculated using the 2^−ΔΔ*Ct*^ method.

### Western blotting

Total proteins were extracted using a NucleoSpin RNA/Protein kit (Macherey Nagel). An equal amount of proteins was reduced, size-separated on 10 or 13.5% SDS-polyacrylamide gels, and transferred to nitrocellulose membranes (Invitrogen). The membranes were blocked in 5% milk in TBS-Tween 0.1% and incubated with specific primary antibodies against CHAC1 (AV42623; Sigma-Aldrich), PARP (9542; Cell Signaling Technology (CST), Danvers, MA, USA), ATF4 (11815; CST), cleaved caspase-3 (9661; CST), phospho- and total NF-κB-p65 (S536 and D14E12; CST), and β-actin (A2228; Sigma-Aldrich). For anti-CHAC1 antibody validation, CHAC1 overexpression was performed with a CHAC1-V5eGFP plasmid (CHAC1-GFP), as previously shown (11). The CHAC1-GFP plasmid was transfected into NCI-H292 cells using Lipofectamine 3000 transfection reagent for 24 h. Secondary antibodies were purchased from CST. Bound antibodies were detected using SuperSignal West Femto Maximum Sensitivity chemiluminescent substrate (Thermo Fisher Scientific, Rockford, IL, USA). Between successive primary antibodies, membranes were treated with Restore PLUS Western Blot Stripping Buffer (Thermo Fisher Scientific). Images were recorded with a Fujifilm LAS-3000 bioimaging system (Fujifilm, Stamford, CT, USA). Quantification was performed with ImageJ 10.2 (https://imagej.nih.gov/ij/index.html).

### ELISA

Concentrations of human IL-8, IL-6, CCL2/MCP-1 (R&D, Minneapolis, MN, USA) and PGE2 (Cayman, Ann Harbor, MI, USA) were measured in cell supernatants using ELISA kits according to the manufacturer's instructions. The 3,3′,5,5′-tetramethylbenzidine substrate was from CST.

### Fluorescence microscopy

CHAC1-GFP expression was monitored in live NCI-H292 cells using an EVOS cell imaging system (Thermo Fisher Scientific).

### NF-κB luciferase activity

NF-κB luciferase activity in A549 cells was assayed with a Luciferase kit (Promega, Madison, WI, USA) according to the manufacturer's protocol with a FLUOstar OPTIMA luminometer (BMG LABTECH, Champigny sur Marne, France).

### Statistical analysis

Differences among groups were assessed for statistical significance using Prism 7.00 software (GraphPad Software, La Jolla, CA, USA) as indicated in the Figure Legends. Differences with *p* < 0.05 were considered statistically significant.

## Results

### Defective regulation of CHAC1 in CF cells

**Figure 1:**
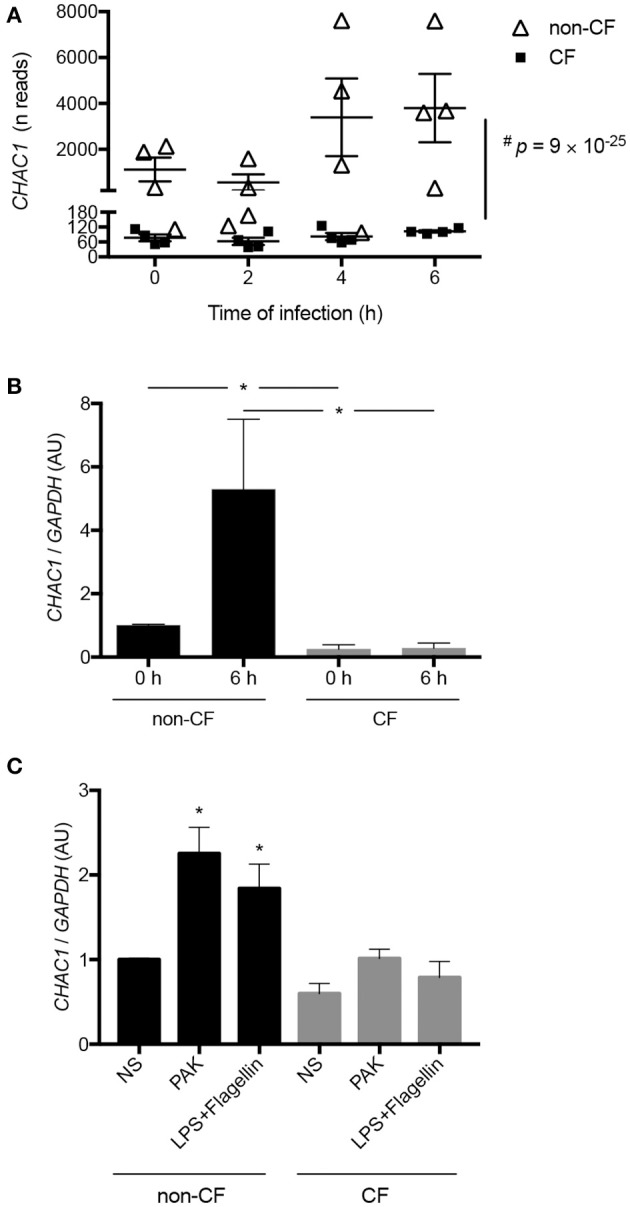
**(A)** Kinetics of *CHAC1* expression measured by RNAseq in hAECBs from patients without CF (*N* = 4, open triangles) and with CF (*N* = 4, black squares) infected with *Pa* (PAK strain, MOI 0.25) for 0, 2, 4, or 6 h (#*p* : Benjamini and Hochberg corrected *p*-value) [data were extracted from (10)]. **(B)** *CHAC1* mRNA expression in hAECBs from patients without CF (*N* = 4, black) and with CF (*N* = 4, gray) infected with *Pa* (PAK strain, MOI 0.25) for 0 or 6 h. Statistical analysis was conducted using Mann-Whitney tests (without vs. with CF groups, *p < 0.05). **(C)** *CHAC1* mRNA expression in hAECBs from patients without CF (black) and with CF (gray) stimulated with 1 μg/mL LPS and 50 ng/mL flagellin or infected with PAK (MOI 0.25) for 6 h. Data are expressed as means ± SEMs of four independent experiments. Statistical analysis was carried out using analysis of variance followed by Dunnett's multiple comparison test (*p < 0.05, NS used as the control group).

In a previous transcriptomic analysis, we identified *CHAC1* as one of the differentially expressed mRNAs between human airway epithelial cells from bronchi (hAECBs) of patients with and without CF during *Pa* infection (10). Indeed, *CHAC1* mRNA was only induced in hAECBs from non-CF patients (Figure 1A, Supplementary Table 1). Because CHAC1 belongs to the UPR pathway of ER stress initiated by PERK (11), we analyzed the expression of genes shown to be involved in CHAC1-associated ER stress responses in our previous study. We observed that *ATF4, PCK2*, and *HERPUD1* were significantly downregulated in hAECBs from CF patients vs. in hAECBs from non-CF patients (Supplementary Figures 1A–C and Supplementary Table 1). In contrast, *ATF3* and *TNFRSF6B* were upregulated (Supplementary Figures 1D,E and Supplementary Table 1) in hAECBs from CF patients during the course of infection by *Pa*. These results indicated an altered expression of mRNAs involved in the CHAC1-associated ER stress response (Supplementary Figure 1F) in CF cells.

We further validated the differential expression of *CHAC1* by qPCR in the same samples used for RNA-seq. Indeed, at baseline, we observed significantly higher expression of *CHAC1* in non-CF cells compared with that in CF cells. As expected from the transcriptomic data, we observed upregulation of *CHAC1* mRNA expression after 6 h of *Pa* infection only in non-CF cells (Figure 1B).

### LPS and flagellin induced CHAC1 overexpression in primary bronchial epithelial cells and NCI-H292 cells

**Figure 2:**
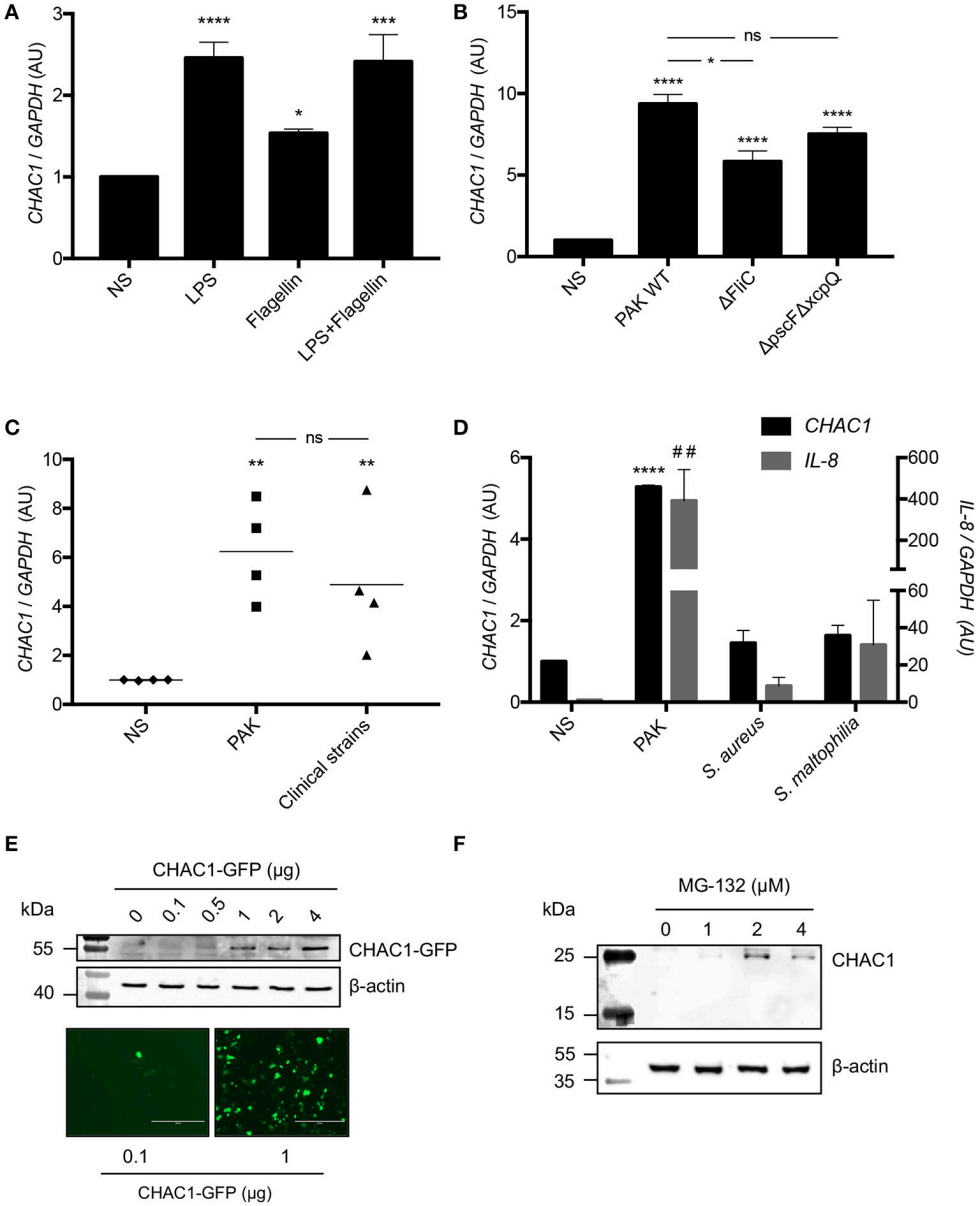
**(A)** *CHAC1* mRNA expression in NCI-H292 cells stimulated with 1 μg/mL LPS and/or 50 ng/mL flagellin for 6 h. Data are expressed as the means ± SEMs of five independent experiments. Statistical analysis was performed using analysis of variance followed by Dunnett's multiple comparison test (control group: NS, *p < 0.05; ***p < 0.001; ****p < 0.0001). **(B)** *CHAC1* mRNA expression in NCI-H292 cells infected with PAK WT, PAK ΔFliC, or PAK ΔpscFΔxcpQ (MOI 0.25) for 6 h. Data are expressed as means ± SEMs of four independent experiments. Statistical analysis was carried out using analysis of variance followed by Bonferroni's multiple comparison test (control group: NS, *p < 0.05; ****p < 0.0001). **(C)** *CHAC1* mRNA expression in NCI-H292 cells infected with PAK strain (MOI 0.25) or different clinical strains of *Pa* (MOI 0.25) for 6 h. Data are expressed as means ± SEMs of four independent experiments (NS, diamonds; PAK, squares; clinical strains, triangles). Statistical analysis was carried out using analysis of variance followed by Bonferroni's multiple comparison test (control group: NS, **p < 0.01). **(D)** *CHAC1* (black) and *IL*-*8* (gray) mRNA expression in NCI-H292 cells infected with PAK, *Staphylococcus aureus* (*S. aureus*), or *Stenotrophomonas maltophilia* (*S. maltophilia*, MOI 0.25) for 6 h. Data are expressed as means ± SEMs of three independent experiments. Statistical analysis was carried out using analysis of variance followed by Dunnett's multiple comparison test (control group: NS, ##*p* < 0.01 [*IL-8*]; ****p < 0.0001 [*CHAC1*]). **(E)** CHAC1 and β-actin protein expression and CHAC1-GFP fluorescence in NCI-H292 cells transfected with increasing amounts of CHAC1-GFP plasmid. **(F)** CHAC1 and β-actin protein expression in NCI-H292 cells treated with increasing concentrations of MG-132 for 6 h.

Because *Pa* induced *CHAC1* expression in non-CF cells, we investigated which *Pa* virulence factors were involved. We studied the effects of two very well-known virulence factors, LPS and flagellin from *Pa*, on hAECBs from CF and non-CF patients and on NCI-H292 cells, which expressed *CHAC1* mRNA at sufficient levels for further knockdown experiments in comparison to BEAS-2B cells (Supplementary Figure 2A). Notably, 6 h of stimulation with LPS and flagellin induced *CHAC1* expression in hAECBs from non-CF patients, similar to whole bacteria at a MOI of 0.25 for 6 h. This induction by LPS and flagellin and by living bacteria was not found in hAECBs from CF patients (Figure 1C). We observed similar effects of LPS and flagellin on *CHAC1* expression in NCI-H292 cells (Figure 2A). To validate the role of flagellin in *CHAC1* expression, we used a mutant of the *Pa* PAK strain, ΔFliC, in which flagellin was deleted. We observed a significantly lower increase in *CHAC1* mRNA expression when NCI-H292 cells were stimulated with the ΔFliC strain than with the WT PAK strain (Figure 2B). We also tested a double mutant of the *Pa* PAK strain with both type II and III secretion systems deleted (ΔpscFΔxcpQ). This mutant induced *CHAC1* mRNA expression, similar to the WT PAK strain (Figure 2B). The difference in CHAC1 expression observed with the ΔFliC strain was not due to different MOI since equal numbers of *Pa* colonies were found on LB agar (Supplementary Figure 3A). Subsequently, in order to determine whether the observed *CHAC1* upregulation was restricted to the laboratory PAK strain, we used *Pa* strains freshly isolated from different patients with CF. We observed that clinical isolates of *Pa* were also able to induce *CHAC1* mRNA expression in NCI-H292 cells (Figure 2C) with an effect similar to PAK. This induction is highly variable between the clinical strains used and may reflect difference of virulence of each strain, since the number of the bacteria used for stimulation is similar (Supplementary Figure 3B). *CHAC1* induction by PAK strain is also highly variable (Figure 2C), and is likely due to the variability in the inoculum used (Supplementary Figure 3B). We also observed that the induction of *CHAC1* mRNA expression by PAK was higher in NCI-H292 cells than in hAECBs (Figures 2B,C vs. Figure 1C). Finally, we tested two other bacterial species known to colonize the airways of patients with CF, i.e., *S. aureus* and *S*. *maltophilia*. Using a similar MOI, we observed reduced *CHAC1* mRNA induction in NCI-H292 cells infected with *S. aureus* and *S. maltophilia* compared with that in the cells infected with *Pa*. Concomitantly, induction of *IL*-*8* mRNA expression was reduced (Figure 2D). The differences in *CHAC1* and *IL-8* mRNA expression observed with *S. aureus* and *S. maltophilia* were not caused by different MOI because equal numbers of colonies were found on LB agar (Supplementary Figure 3C).

Next, we examined CHAC1 protein expression in response to *Pa* stimulation. Consistent with previous reports indicating that CHAC1 protein is very unstable due to its rapid degradation by the proteasome in Neuro2a cells (23), we were unable to detect endogenous CHAC1 protein in NCI-H292 at baseline (Supplementary Figure 2B) or after *Pa* infection (data not shown). Additionally, we were unable to detect CHAC1 protein expression after treating the cells with tunicamycin, a known inducer of *CHAC1* mRNA expression (24) (data not shown). CHAC1 was only detected at the protein level when it was either overexpressed with a plasmid in NCI-H292 cells (Figure 2E) or when the cells were treated with at least 1 μM of the proteasome inhibitor MG-132 (Figure 2F).

### CHAC1 upregulation was ATF4 dependent and PERK independent

**Figure 3:**
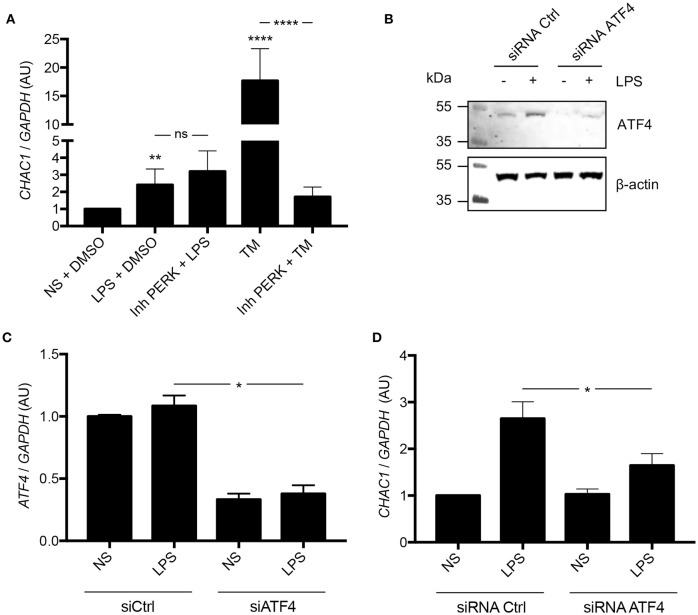
**(A)** *CHAC1* mRNA expression in NCI-H292 cells stimulated with 1 μg/mL LPS in DMSO (vehicle) or 1 μM PERK inhibitor (Inh PERK) or stimulated with 0.5 μg/mL tunicamycin (TM) with or without PERK inhibitor for 6 h. Data are expressed as means ± SEMs of five independent experiments. Statistical analysis was performed with analysis of variance followed by Bonferroni's multiple comparison test (**p < 0.01, ****p < 0.0001). **(B)** Western blot analysis of ATF4 expression in NCI-H292 cells treated 48 h with siRNA Ctrl or siRNA ATF4 and stimulated with (+) or without (–) LPS for 1 h (images are representative of six independent experiments). **(C)** *ATF4* mRNA expression in NCI-H292 cells treated for 48 h with siRNA Ctrl or siRNA ATF4 and stimulated with or without (NS) LPS for 1 h. Data are means ± SEMs of six independent experiments. Statistical analysis was performed using Mann-Whitney tests (*p < 0.05). **(D)** *CHAC1* mRNA expression in NCI-H292 cells treated for 48 h with siRNA Ctrl or siRNA ATF4 and stimulated with or without (NS) LPS for 1 h. Data are means ± SEMs of six independent experiments. Statistical analysis was performed using Mann-Whitney tests (*p < 0.05).

Because CHAC1 has been shown to be associated with the PERK pathway (11), we hypothesized that induction of *CHAC1* by LPS from *Pa* may be mediated by PERK. Therefore, we used a PERK inhibitor and tunicamycin (TM) as a positive control for activation of the CHAC1 and PERK pathway (11). As expected, compared with untreated NCI-H292 cells, LPS and TM induced *CHAC1* mRNA expression. When cells were treated with TM and PERK inhibitor, there was a significant 90.3% decrease in *CHAC1* mRNA expression compared with that in cells treated with TM alone (Figure 3A). However, when cells were treated with LPS and PERK inhibitor, *CHAC1* mRNA expression was not significantly different from that in cells treated with LPS only (Figure 3A). We then hypothesized that this induction of *CHAC1* by LPS would directly pass through ATF4. Indeed, we observed that LPS was able to activate ATF4 and that siRNA against *ATF4* inhibited this induction (Figure 3B). The inhibition of ATF4 expression by the siRNA ATF4 (~60%) was associated with an inhibition of CHAC1 induction by LPS (Figures 3C,D).

### CHAC1 overexpression induced by *Pa* did not stimulate apoptosis

**Figure 4:**
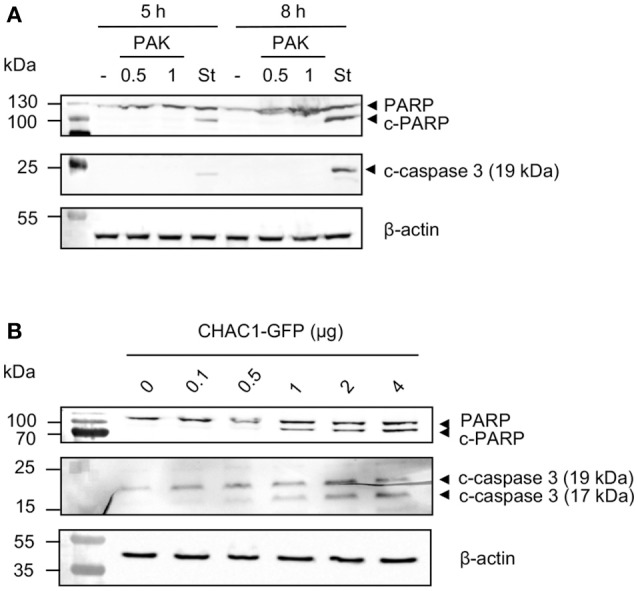
**(A)** PARP (uncleaved form), c-PARP (cleaved form), c-caspase 3 (cleaved form), and β-actin protein expression in NCI-H292 cells infected with PAK (MOI 0.5 or 1) or stimulated with 1 μM staurosporine (St) for 5 or 8 h. **(B)** PARP (uncleaved form), c-PARP (cleaved form), c-caspase 3 (cleaved forms) and β-actin protein expression in NCI-H292 cells transfected with p-CHAC1-GFP (same blot as shown in Figure 2E).

CHAC1 has been shown to play a role in apoptosis (11); therefore, we investigated the effects of *Pa* stimulation on apoptosis. We infected NCI-H292 cells with two different MOIs (0.5 and 1) for 5 or 8 h and examined the levels of cleaved PARP and cleaved caspase-3. As expected, we observed that the positive control staurosporine induced cleavage of PARP and caspase-3. In contrast, *Pa* did not induce cleavage of PARP and caspase-3 (Figure 4A). PARP and caspase-3 cleavages were observed when CHAC1 was overexpressed with a plasmid in NCI-H292 cells (Figure 4B).

### CHAC1 regulated the inflammatory response of bronchial epithelial cells induced by LPS or flagellin

**Figure 5:**
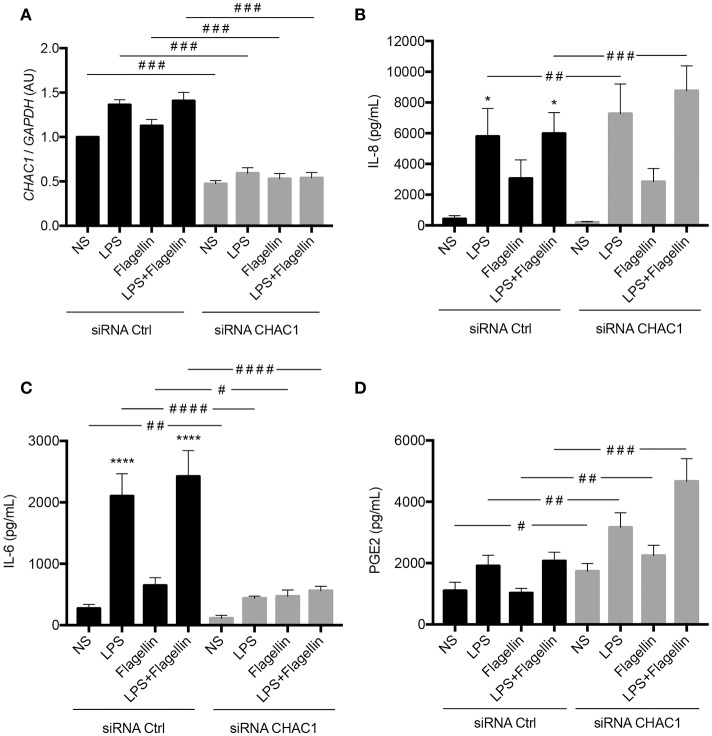
**(A)** *CHAC1* mRNA expression in NCI-H292 cells transfected with siRNA control (siRNA Ctrl, black) or siRNA CHAC1 (gray) for 48 h and stimulated with LPS and/or flagellin for 6 h. Data are expressed as means ± SEMs of 10 independent experiments. **(B)** IL-8, **(C)** IL-6, and **(D)** PGE_2_ productions in supernatants from cells transfected with siRNA control (siRNA Ctrl, black) or siRNA CHAC1 (gray) for 48 h and stimulated with LPS and/or flagellin for 6 h. Data are expressed as means ± SEMs of 10 independent experiments. Statistical analysis was performed using analysis of variance followed by Dunnett's multiple comparison tests (control group: NS, *p < 0.05, ****p < 0.0001) and Wilcoxon tests (siRNA Ctrl vs. siRNA CHAC1 groups, ^#^*p* < 0.05, ^##^*p* < 0.01, ^###^*p* < 0.001, ^####^*p* < 0.0001).

CHAC1 is a component of the ER stress pathway, and a link between ER stress and the inflammatory response has already been described in the context of CF (12). Therefore, we investigated the role of CHAC1 in the inflammatory response induced by LPS or flagellin. Transfection with *CHAC1* siRNA induced around 50% reduction in *CHAC1* mRNA expression (Figure 5A). We then examined different inflammatory parameters, particularly the secretion of IL-8, IL-6, and PGE2. As expected, we observed that 1 μg/mL LPS, 50 ng/mL flagellin, and the combination of both (LPS+flagellin) induced significant increases in both IL-8 and IL-6 secretion and tended to increase PGE_2_ secretion (Figures 5B–D). Under these stimulation conditions, when *CHAC1* mRNA expression was decreased using siRNA, greater significant increases in IL-8 secretion (Figure 5B) and PGE_2_ secretion (Figure 5D) were observed. In contrast, in the presence of *CHAC1* siRNA, we observed significant inhibition of IL-6 secretion (Figure 5C) in cells stimulated with LPS (80% inhibition), flagellin (30% inhibition), or the combination of both virulence factors (80% inhibition). We obtained similar results for the mRNA expression of *IL-8* and *IL-6* (Supplementary Figures 4A,C). *CCL2* mRNA expression was also modulated similar to *IL-8* expression under these experimental conditions (not detectable by ELISA; Supplementary Figure 4B).

### CHAC1 regulated NF-κB activation

**Figure 6:**
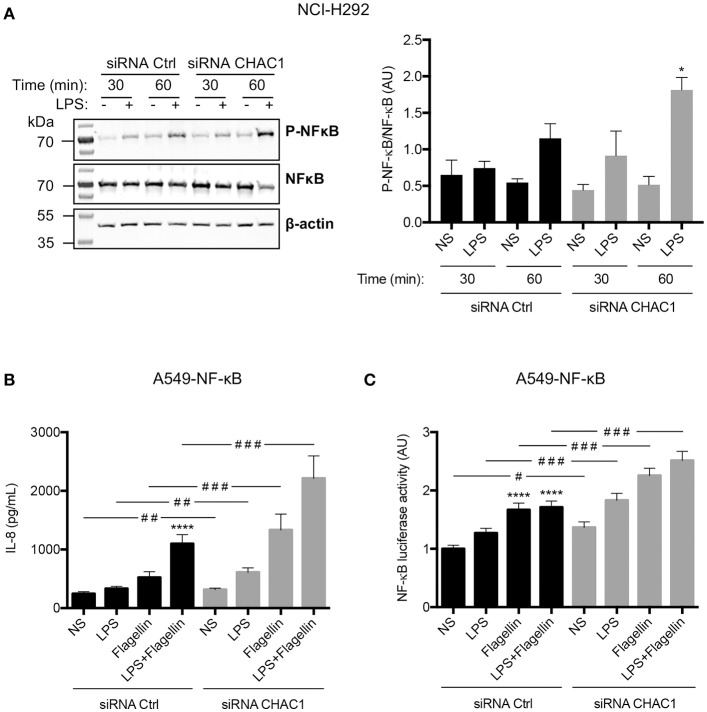
**(A)** Phospho-NF-κB (p-NF-κB), total NF-κB (NF-κB), and β-actin protein expression in NCI-H292 cells transfected with siRNA control (siRNA Ctrl) or siRNA CHAC1 for 48 h and then stimulated for 30 min or 1 h with (+) or without (–) 1 μg/mL LPS (images are representative of four independent experiments; left panel). Results are shown as p-NF-κB/NF-κB ratios (right panel). Data are expressed as means ± SEMs of four experiments. Statistical analysis was carried out using Mann-Whitney tests (*p < 0.05, siCtrl-LPS 1 h vs. siCHAC1-LPS 1 h). **(B)** IL-8 production and **(C)** NF-κB luciferase activity in A549-NF-κB luciferase cells transfected with siRNA control (siRNA Ctrl, black) or siRNA CHAC1 (gray) for 48 h and stimulated with LPS and/or flagellin for 24 h. Data are expressed as means ± SEMs of four independent experiments. Statistical analysis was performed using analysis of variance followed by Dunnett's multiple comparison tests (control group: NS, ****p < 0.0001) and Wilcoxon test (siRNA Ctrl vs. siRNA CHAC1 groups, ^#^*p* < 0.05; ^##^*p* < 0.01; ^###^*p* < 0.001).

To determine whether regulation of these inflammatory parameters involved NF-κB, we studied its phosphorylation by western blot in NCI-H292 cells. We observed that LPS induced a significant increase in NF-κB p65 phosphorylation in *CHAC1*-knockdown cells after 1 h of stimulation (Figure 6A). To confirm these data, we used A549-NF-κB luciferase cells. As in NCI-H292 cells, 60% inhibition of *CHAC1* mRNA expression was observed after siRNA transfection (data not shown). Similar to NCI-H292 cells, we observed that 1 μg/mL LPS, 50 ng/mL flagellin, and the combination of both (LPS+flagellin) induced a significant increase in IL-8 secretion when A549 cells were transfected with *CHAC1* siRNA compared with that in cells transfected with siRNA control (Figure 6B). Additionally, as expected, we observed that 1 μg/mL LPS, 50 ng/mL flagellin, and the combination of both (LPS+flagellin) induced significant increases in NF-κB activity (Figure 6C). Under these experimental conditions, when we decreased *CHAC1* mRNA expression, we observed an even greater increase in NF-κB activity when cells were stimulated with LPS and/or flagellin (Figure 6C). IL-6 and CCL-2 were not detectable by ELISA (data not shown).

## Discussion

Independent of *CFTR* mutations, links between ER stress and CF-related inflammation have been extensively studied, particularly with regard to inositol-requiring enzyme 1 (IRE1α), and PERK (25) pathways. In our previous study, we identified the *CHAC1* mRNA as one of the most highly differentially expressed transcripts between primary bronchial cells from CF and non-CF patients, infected with *Pa*. We also found that CF cells exhibited an expected excessive inflammatory response, including higher IL-8 production (10). Because CHAC1 was recently described as a new component of the UPR in the PERK pathway (12), we aimed to evaluate whether CHAC1 was involved in the modulation of the inflammatory response induced by *Pa* in lung epithelial cells.

We confirmed our previous transcriptomic data showing that *CHAC1* mRNA was overexpressed in primary non-CF epithelial cells compared with its expression in CF cells at baseline and upon *Pa* infection. Additionally, *Pa* and two of its virulence factors, LPS and flagellin, the respective ligands of Toll-Like Receptor (TLR) 4 and TLR5, were able to induce *CHAC1* at the transcriptional level in bronchial epithelial NCI-H292 cells. As previously shown in Neuro2a (23) or HEK cells (26), we were able to detect CHAC1 protein expression only after MG-132 treatment, suggesting the involvement of the proteasome pathway in the stability of the protein (26). This induction of *CHAC1* mRNA expression was observed not only with the laboratory strain PAK but also with freshly isolated clinical strains. Interestingly, *S. maltophilia* and *S. aureus*, both known to chronically infect CF patients, were not able to induce *CHAC1* mRNA expression to similar levels. Using cellular infection models, other transcriptomic studies have consistently shown *CHAC1* upregulation with microorganisms, including human coronavirus (27), human cytomegalovirus (28), tick-borne flaviviruses (29), Zika virus (30), and the bacterium *Mycoplasma hominis* (31). In these studies, the specific role of CHAC1 was not investigated. However, CHAC1 is essential as embryonic lethality was observed in mice with deletion of the *Chac1* gene (32).

In our study, *CHAC1* mRNA induction by LPS and *Pa* was not related to PERK activation, as is TM, but was dependent on ATF4. In contrast, LPS from *E. coli* has previously been shown to activate both PERK and ATF4 in A549 cells (33). Notably, ATF4 has been shown to be directly recruited by TLR4 independent of ER stress in human monocytes (34). Consistent with our results, another ER stress-associated molecule, GADD34, was recently shown to be upregulated by several virulence factors from *Pa*, including pyocyanin and AprA, independent of PERK but dependent on ATF4 (35). Taken together, these results were consistent with emerging evidence showing that induction of the UPR pathway by microorganisms did not reflect ER stress but was an integral part of the immune response and TLR pathways (36).

The fine tuning of immune responses by the UPR has been extensively studied (37). However, its contribution to the regulation of cytokine production in bacterial infection is not well understood (16). Here, we observed that inhibition of *CHAC1* mRNA expression by siRNA promoted IL-8 and CCL2 expression and PGE2 secretion, suggesting that CHAC1 prevents excessive inflammatory responses dependent on the NF-κB p65 pathway. These results were consistent with a recent study reporting that bronchial epithelial cells undergoing ER stress showed reduced IL-8 and CXCL1 production in the presence of inflammatory stimuli (38). Indeed, pretreatment of bronchial epithelial cells with TM [shown here and by others (11) to be able to increase CHAC1 expression] can prevent IL-8 and CXCL1 production. In contrast, inhibition of *CHAC1* mRNA expression by siRNA inhibits IL-6 production, suggesting that CHAC1 may promote IL-6 expression. Our results were consistent with a previous study showing that TM promotes IL-6 secretion in bronchial IB3-1 (CF deficient) and C38 cells stimulated with flagellin (12). Previous works have demonstrated that IL-6 production in A375 skin cells is dependent on CHOP (39). However, CHOP is expected to be upstream in the CHAC1 pathway and does not explain our results; future studies are needed to determine how the IL-6 pathway is affected specifically in lung epithelial cells.

In addition to its role in UPR, CHAC1, as a member of the γ-glutamylcyclotransferase family, is able to hydrolyze glutathione only in its reduced form (GSH) (40). Glutathione has been widely studied owing to the function of CFTR as a glutathione transporter (41). GSH homeostasis is defective in CF, and GSH promotes oxidative stress and related inflammation (42). The roles of CHAC1 activity in relation to cellular glutathione homeostasis and the observed cytokine regulation remain to be determined, although previous studies have demonstrated that the levels of glutathione-related enzyme can regulate cytokine production (43).

In our experiments, PAK did not induce apoptosis in NCI-H292 cells. However, these cells are not completely resistant to apoptosis since staurosporine was able to induce caspase-3 and PARP cleavage. Thus, although *CHAC1* was induced by *Pa*, it was not sufficient to induce cell apoptosis. It is conceivable that the level of induction of *CHAC1* may be too low to promote apoptosis. In contrast, high induction of CHAC1 using an overexpression plasmid led to induction of PARP cleavage, indicative of cell apoptosis. Apoptosis dysfunction is still highly controversial in CF (44), and our results are partly consistent with some previous studies. Indeed, even if *Pa* has been previously shown to induce apoptosis in lung epithelial cells, variable susceptibility between lung epithelial cells has been observed. Moreover, the PAO1 strain of *Pa* has been shown to induce apoptosis in human tracheal 9HTEo cells but not in primary nasal epithelial cells from polyps or in 16HBE14o bronchial cells, which are both resistant (45). In contrast, other bronchial cell lines are permissive to apoptosis induced by *Pa*, such as IB3-1 (F508del/W1282X), C38, S9 (both IB3-1 corrected cells), CFT1-LCFSN, and CFT1-LC3 (F508del) cells. More recently, using Calu-3 polarized lung epithelial cells, researchers showed that PAO1 was able to induce apoptosis markers, such as active caspase-3, within 6–8 h. Additionally, the effects of the *CFTR* genotype on apoptosis of lung epithelial cells at baseline (44) or after *Pa* infection are still debated and may be dependent on which specific marker of apoptosis is being evaluated (45, 46).

In conclusion, we showed that inhibition of *CHAC1* in bronchial epithelial cells resulted in the regulation of inflammatory mediators. Moreover, *CHAC1* was induced preferentially in normal hAECBs in comparison to CF hAECBs in response to *Pa* and its virulence factors, LPS and flagellin. Thus, these results suggest that low CHAC1 expression may contribute to the excessive inflammation observed in CF bronchial epithelial cells after *Pa* infection and thus may be associated with the exacerbation of inflammation characterizing the lungs from patients with CF. Inflammation is complex and thought to have a critical impact during the course of CF lung disease (47). A better understanding of the link between infection and inflammation is thus required for the development of new therapeutics.

## Author contributions

LG, MC, and VB designed the study. LG and LP wrote the manuscript. LP, VB, TF, and HP performed the experiments. LG, LP, and VB performed the data analysis. LT participated in PGE_2_ and *Staphylococcus* experiments. DM participated in bacterial sample collection. IM, DM, LT, MC, HC, and VB critically revised the manuscript.

### Conflict of interest statement

The authors declare that the research was conducted in the absence of any commercial or financial relationships that could be construed as a potential conflict of interest.
