# Supplementary figures and images for: CHAC1 Is Differentially Expressed in Normal and Cystic Fibrosis Bronchial Epithelial Cells and Regulates the Inflammatory Response Induced by Pseudomonas aeruginosa
Source: Front Immunol. 2018 Nov 29;9:2823. doi: 10.3389/fimmu.2018.02823 (PMC6282009; doi:10.3389/fimmu.2018.02823)

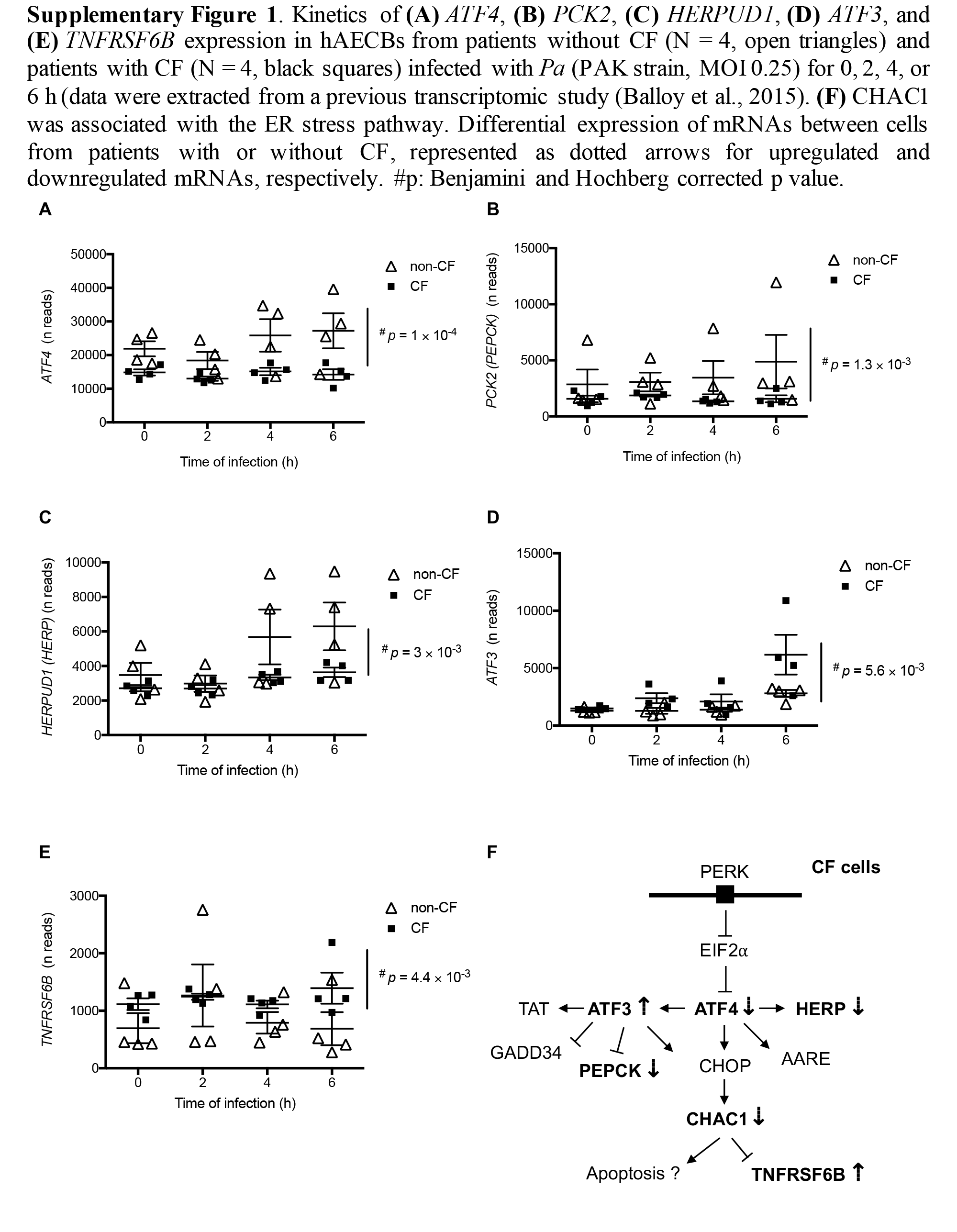

Supplement: Supplementary file 1 [file Image_1.tif]

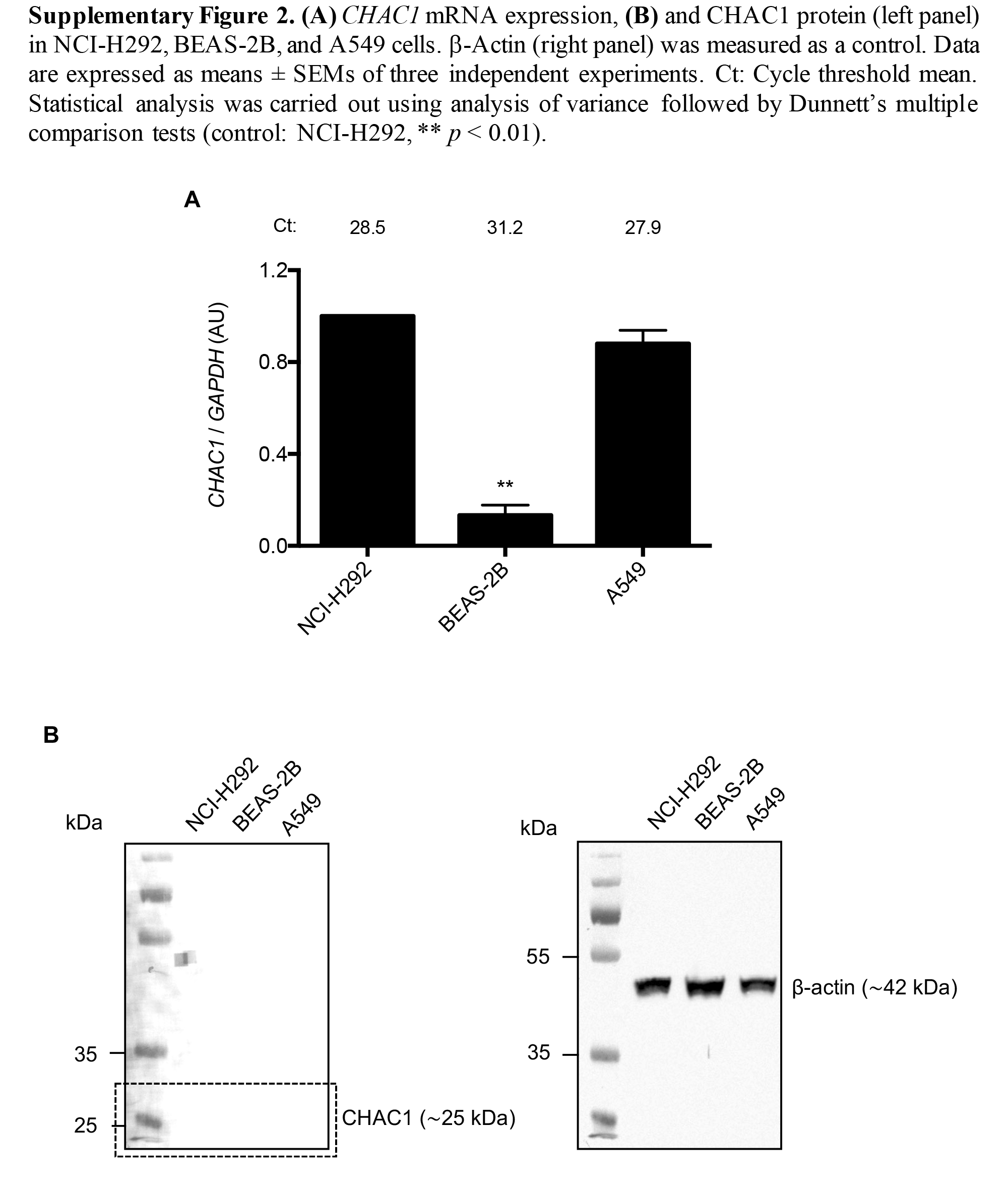

Supplement: Supplementary file 2 [file Image_2.tif]

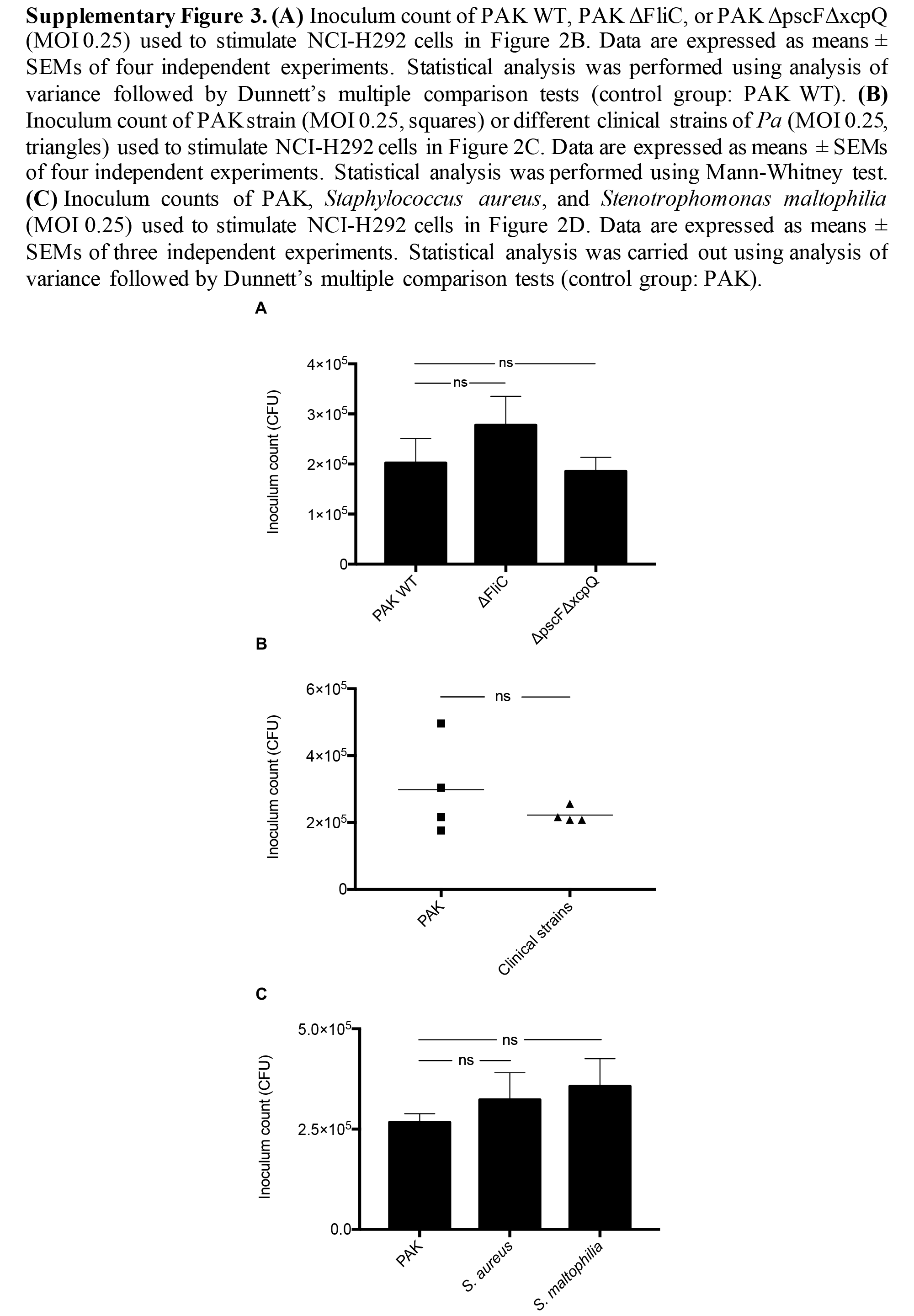

Supplement: Supplementary file 3 [file Image_3.TIF]

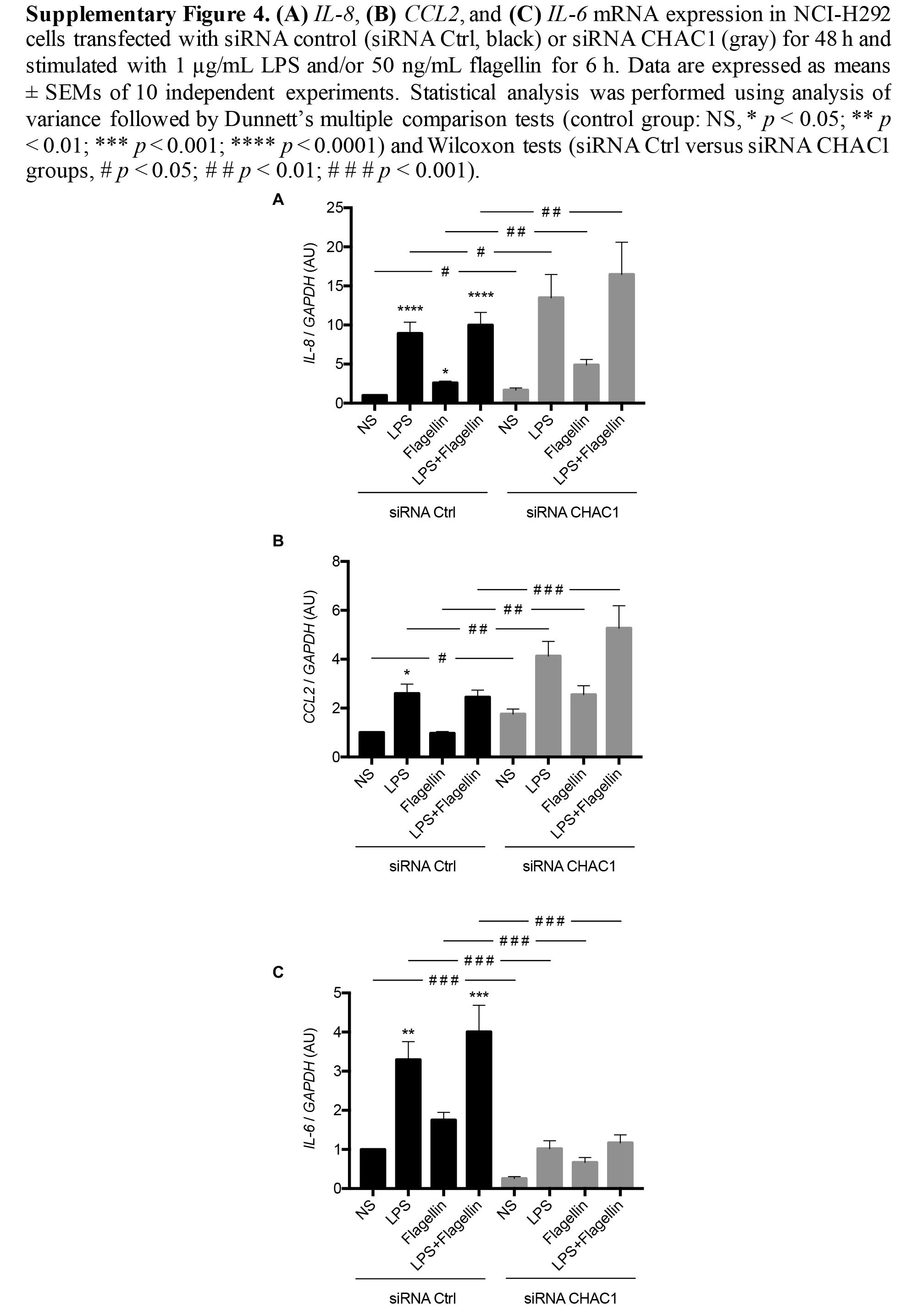

Supplement: Supplementary file 4 [file Image_4.TIF]
