# Supplementary material for: CHAC1 Is Differentially Expressed in Normal and Cystic Fibrosis Bronchial Epithelial Cells and Regulates the Inflammatory Response Induced by Pseudomonas aeruginosa
Source: Front Immunol. 2018 Nov 29;9:2823. doi: 10.3389/fimmu.2018.02823 (PMC6282009; doi:10.3389/fimmu.2018.02823)
Supplement: Supplementary file 5 [file Table_1.docx]

|  | **0 h (CF/non-CF)** | | **2 h (CF/non-CF)** | | **4 h (CF/non-CF)** | | **6 h (CF/non-CF)** | |
| --- | --- | --- | --- | --- | --- | --- | --- | --- |
|  | **Log2FC** | ***p* (adjBH)** | **Log2FC** | ***p* (adjBH)** | **Log2FC** | ***p* (adjBH)** | **Log2FC** | ***p* (adjBH)** |
| *DDIT3*, ENSG00000175197 |  |  |  |  |  |  |  |  |
| ***ATF4*, ENSG00000128272** | -0.55 | 3.03 E-02 |  |  | -0.70 | 6.15 E-03 | -0.87 | 1.47 E-04 |
| ***ATF3*, ENSG00000162772** |  |  | 0.84 | 2.98 E-02 |  |  | 0.99 | 5.64 E-03 |
| *HSPA5*, ENSG00000044574 |  |  |  |  |  |  |  |  |
| **TNFRSF6B, ENSG00000243509** |  |  |  |  |  |  | 1.24 | 4.47 E-03 |
| EIF2A, ENSG00000144895 |  |  |  |  |  |  |  |  |
| EIF2AK3, ENSG00000172071 |  |  |  |  |  |  |  |  |
| CCND1, ENSG00000110092 |  |  |  |  |  |  |  |  |
| ***PCK2*, ENSG00000100889** |  |  |  |  | -1.04 | 1.70 E-02 | -1.24 | 1.35 E-03 |
| FBP1, ENSG00000165140 |  |  |  |  |  |  |  |  |
| ***CHAC1*, ENSG00000128965** | -3.24 | 6.02 E-18 | -2.70 | 3.71 E-08 | -4.29 | 1.82 E-22 | -4.49 | 9.27 E-25 |
| PPP1R15A, ENSG00000087074 |  |  |  |  |  |  |  |  |
| NFE2L2, ENSG00000116044 |  |  |  |  |  |  |  |  |
| ***HERPUD1*, ENSG00000051108** |  |  |  |  | -0.60 | 2.05 E-02 | -0.69 | 3.04 E-03 |

**Supplementary Table 1.**

**Supplementary Table 1**. Expression of ER stress response mRNAs was measured by RNA-seq analysis in primary hAECBs from patients with CF (N = 4) and without CF (N = 4) during infection by *Pa* from 0 to 6 h (PAK strain, MOI 0.25; log2FC: CF versus non‑CF cells; data were extracted from (Balloy et al., 2015). The shaded lines are those for which the results were statistically significant. Log2FC: Log2 Fold change; P (adjBH): Benjamini and Hochberg corrected p value.
